# A Cell Culture Adapted HCV JFH1 Variant That Increases Viral Titers and Permits the Production of High Titer Infectious Chimeric Reporter Viruses

**DOI:** 10.1371/journal.pone.0044965

**Published:** 2012-09-13

**Authors:** Shuanghu Liu, Li Xiao, Cassie Nelson, Curt Hagedorn

**Affiliations:** 1 Departments of Medicine, School of Medicine, University of Utah, Salt Lake City, Utah, United States of America; 2 Experimental Pathology, School of Medicine, University of Utah, Salt Lake City, Utah, United States of America; Pohang University of Science and Technology, Republic of Korea

## Abstract

The unique properties of the hepatitis C virus (HCV) JFH1 isolate have made it possible to produce and study HCV in an infectious cell culture system. However, relatively low virus titers restrict some of the uses of this system and preparing infectious chimeric reporter viruses have been difficult. In this study, we report cell culture-adapted mutations in wild-type JFH1 yielding higher titers of infectious particles of both JFH1 and chimeric JFH1 viruses carrying reporter genes. Sequencing analyses determined that ten of the sixteen nonsynonymous mutations were in the NS5A region. Individual viruses harboring specific adaptive mutations were prepared and studied. The mutations in the NS5A region, which included all three domains, were most effective in increasing infectious virus production. Insertion of two reporter genes in JFH1 without the adaptive mutations ablated the production of infectious HCV particles. However, the introduction of specific adaptive mutations in the NS5A region permitted reporter genes, Renilla luciferase (Rluc) and EGFP, to be introduced into JHF1 to produce chimeric HCV-NS5A-EGFP and HCV-NS5A-Rluc reporter viruses at relatively high titers of infectious virus. The quantity of hyperphosphorylated NS5A (p58) was decreased in the adapted JFH1 compared wild type JFH1 and is likely be involved in increased production of infectious virus based on previous studies of p58. The JFH1-derived mutant viruses and chimeric reporter viruses described here provide new tools for studying HCV biology, identifying HCV antivirals, and enable new ways of engineering additional infectious chimeric viruses.

## Introduction

The hepatitis C virus (HCV) is a single-stranded, positive-sense RNA virus of the genus Hepacivirus within the family Flaviviridae [1], [2]. A 5′ internal ribosome entry site (IRES) drives RNA translation to produce a polyprotein of approximately 3,000 amino acids (aa) that encodes both structural and nonstructural proteins [3]–[5]. HCV is a major cause of chronic hepatitis, liver cirrhosis and hepatocellular carcinoma [6], [7]. More than 170 million people worldwide are chronically infected with HCV [8]. Current treatments are effective in generally less than half of patients completing 48 weeks of therapy and new combination antiviral therapies are needed [9], [10].

Studies of the HCV life cycle and virus-host interactions have been hampered by the lack of a high titer infectious cell culture system and small-animal models of HCV infection [5], [11]. The development of HCV replicon systems was a major advance in studying aspects of HCV replication, viral protein processing and viral-host cell interactions [12], [13]. A major breakthrough was made by establishing an infectious HCV cell culture system using a genotype 2a isolate (JFH1 strain) of HCV and Huh-7 cells [14]–[16]. In this system infectious HCV particles are secreted in an envelope glycoprotein-dependent manner and enable a variety of questions to be answered regarding HCV biology and cell infection. However, virus titers of JFH1 released from infected cells are relatively low compared to a number of other (+) stranded RNA virus cell culture systems and limit some of the applications of this system. Recent studies have identified adaptive or compensatory mutations that enhance infectious virus particle production from either wild-type JFH1 or intergenotypic chimeras [15], [17]–[23]. Although these mutations have been found in nearly all of the structural and nonstructural proteins, the majority have mapped to the core-NS2 coding region, with a noticeable clustering in the p7 and NS2 proteins [21], [22]. The functions of p7 and NS2 are not well defined, but evidence is accumulating that these proteins may be involved in assembly and/or release of infectious virus particles [24], [25], [26], and that adaptive mutations in p7 and NS2 improve their functions in virus assembly or release [22]. Enhancement in infectious viral titers has also been attributed to adaptive mutations in the E2 glycoprotein [18], [19] and NS5A protein [17].

NS5A is an RNA binding phosphoprotein composed of three domains that are separated by trypsin-sensitive low-complexity sequences (LCS I and LCS II) and an N-terminal amphipathic alpha-helix that anchors the protein in intracellular membranes [27], [28]. An X-ray crystallography structure of domain I showed a dimer with a claw like shape that can bind a single-stranded RNA molecule [29]. Domain III of NS5A plays an important role in virus assembly and the production of infectious particles [30]–[36]. NS5A exists as two phosphorylated forms, migrating as 56 and 58 kDa proteins on SDS-PAGE gels [60], [61]. The two major phospho-isoforms are also referred to as basally phosphorylated and hyperphosphorylated, respectively. Previous studies showed that adaptive mutations which reduce NS5A hyperphosphorylation increase HCV replication and production of virus. Studies of subgenomic HCV replicons have provided evidence that high amounts of NS5A-p58 inhibit HCV replication in cell culture (62). Several cell culture adaptive mutations in NS5A have been reported to increase the production of infectious HCV particles. They include a V2440L adaptive mutation which slows cleavage at the NS5A-NS5B site and increases the production of infectious virus [17] and a T2438I mutation that enhances the production of a JFH1-NS5A-EGFP reporter virus [37]. Nevertheless, adaptive mutations in the NS5A region that produce major increases in the titer of infectious JFH1 are relatively infrequent as compared to adaptive mutations in the core-NS2 region and none have been reported that allow the insertion of reporter genes into the NS5A genomic region that generally ablate or decrease the production of infectious chimeric viruses. Enhancing the production of infectious JFH1, particularly chimeric reporter viruses, has a number of practical applications in advancing studies of HCV biology and identifying new antivirals.

In this study we identified an adaptive variant of wild-type HCV JFH1, following serial passages of virus in Huh-7.5 cells, that increased the production of infectious HCV particles by at least two logs. Whole genome sequencing analyses showed that most of the mutations in this adapted virus were in the NS5A region. Comparing the production of infectious recombinant viruses with selected mutations revealed that the NS5A protein of HCV was the major element modifying the production of infectious viral particles. Mutations in all three domains of NS5A enhanced infectious virus production. The introduction of these NS5A mutations into wild-type JFH1 allowed reporter genes (EGFP and *Renilla* luciferase) to be inserted into the NS5A genomic region and relatively high titer infectious HCV chimeric reporter viruses to be produced.

## Methods

### Cell Culture

Huh-7.5 cells, a human hepatoma cell line, were generously provided by Dr. Charles M. Rice [38]. Cells were maintained in Dulbecco’s modified Eagle’s medium (DMEM) (Invitrogen) supplemented with 100 U/ml of penicillin, 100 µg/ml of streptomycin, nonessential amino acids, and 10% fetal bovine serum (FBS) (Invitrogen) at 37°C in 5% CO_2_. All experiments described in this study were performed using these cells.

### Antibodies

The monoclonal antibody to the NS5A protein was a gift from Dr. Chen Liu (University of Florida, Gainesville, FL) and the secondary goat anti-mouse IgG conjugated with Alexa Fluor 594 was purchased from Invitrogen.

### Plasmids

Plasmid constructs were based on the consensus sequence of HCV pJFH1 which was kindly provided by Dr. Wakita [39]. Amino acid substitutions were introduced by overlapping extension PCR. All new clones were sequenced using an ABI PRISM 3100-Avant Genetic Analyzer (Applied Biosystems) [40]. Full length clones with the correct sequences documented were used for experiments.

### Generation of Cell Culture-adapted High Titer JFH1 Virus During Serial Passage

**Figure 1:**
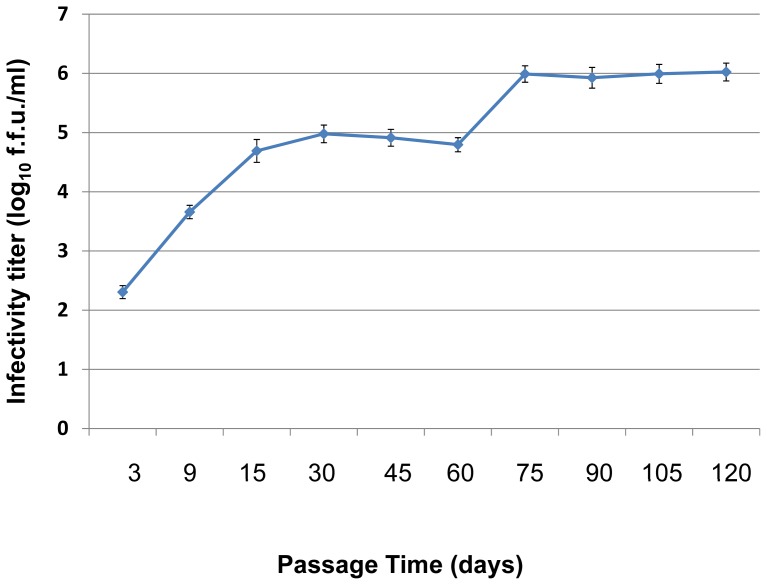
The generation of high titer cell culture-adapted JFH1 virus by serial passages. To produce high titer cell culture adapted infectious JFH1 HCV, *in vitro* transcribed, JFH1-wt RNA was electroporated into Huh-7.5 cells. Transfected Huh-7.5 cells were maintained and passaged every three days. Five passages were defined as one cycle. Culture supernatant at the end of each cycle was used to infect fresh Huh-7.5 cells. A total of 8 cycles (40 passages or 120 days) were done. During the adaptation process, the titer of infectious HCV was determined by immunofluorescence assays. The viral titer is expressed as focus-forming units per milliliter (f.f.u./ml) measured in triplicate. The data are presented as mean ± standard deviations (n = 6).

To produce adapted high titer infectious HCV, *in vitro* transcribed JFH1-wt RNA was electroporated into Huh-7.5 cells. Transfected Huh-7.5 cells were maintained and subcultured (passaged) every three days. Five passages were defined as one cycle. The culture supernatant from the last (5^th^) passage of each cycle was used to infect fresh Huh-7.5 cells. A total of 8 cycles and 40 passages (120 days) were performed. During the adaptation process, the titer of infectious HCV was determined by immunofluorescence assays. The viral titer is expressed as focus-forming units per milliliter (f.f.u./ml) in triplicate. At day 75 the virus titer reached 1.1x10^6^ f.f.u./ml. Infected cells were passaged for another 45 days (3 cycles). The virus titer of these cultures was approximately 1×10^6^ f.f.u./ml and was used for subsequent experiments (Figure 1).

### Construction of the JFH1 Adaptive Variant (JFH1-AM120) cDNA

Total RNA was isolated from a 6-cm-diameter dish of confluent Huh-7.5 cells infected with the 120^th^ day adapted virus population by Trizol (Invitrogen). The total RNA was reverse transcribed by using Superscript III Reverse Transcriptase (Invitrogen) and random primers. The resulting cDNA was used as a template for subsequent PCR with Platinum® Pfx DNA polymerase (Invitrogen) and the following four pairs of primers according to the manufacturer’s instructions:

JFH1-For, 5′- ACCTGCCCCTAATAGGGGCGACACT-3′;

JFH1-3059-Rev, 5′-TTAAGAGGTAAGCAGGCCCAAGCA-3′;

JFH1-2880-For, 5′-GTGGTGGTTGTGCTATCTCCTGACCCT-3′;

JFH1-5400-Rev, 5′-TGATGGAAACGCATCCAGTCGCCA-3′;

JFH1-5233-For, 5′-AATGAGGTCACCCTCACACA-3′;

JFH1-7816-Rev, 5′- GATGTTGTACAGTACACCTTG -3′;

JFH1-7645-For, 5′- GTCTTGGTCTACTTGCTC-3′; and.

JFH1-9678-Rev, 5′-CCTGCAGGTCGACTCTAGACATGA-3′.

**Figure 2:**
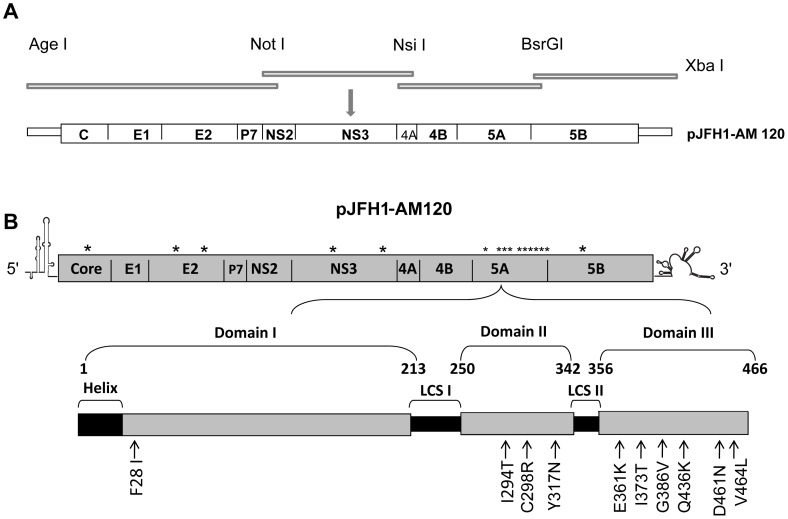
Sequence analysis of the adapted HCV JFH1 variant (JFH1-AM120) and construction of pJFH1-AM120. (Panel A).Total RNA was isolated from confluent Huh-7.5 cells infected with the 120^th^ day adapted virus (Methods). The total RNA was reverse transcribed with random primers and the resulting cDNA was used as a template for subsequent PCR amplification of four overlapping amplicons (Panel A) (Methods). Four fragments of HCV cDNA (nt1–3059, 2880–5400, 5233–7816 and 7645–9678) were amplified to cover the entire HCV genome. The PCR amplicons were sequenced and the four fragments were sequentially sub-cloned into pJFH1 using the unique restriction enzyme sites Age I/Not I, Not I/Nsi I, Nsi I/BsrG I and BsrG I/Xba I. The plasmid encoding the adapted pJFH1 variant was designated JFH1-AM120 (Panel B). Location of adaptive mutations in JFH1-AM120 are indicated by *.

The four PCR products of HCV cDNA (NT1–3059, 2880–5400, 5233–7816 and 7645–9678) were amplified to cover all of the HCV genome. The PCR amplicons were analyzed by 1% agarose gel electrophoresis and sequenced as described previously [40]. The four fragments were sequentially subcloned into pJFH1 using the unique restriction enzyme sites Age I/Not I, Not I/Nsi I, Nsi I/BsrG I and BsrG I/Xba I, respectively, to produce JFH1-AM120 which contained all sixteen nonsynonymous mutations found in the adapted virus after 120 days of passage. A JFH1-wt plasmid was used to prepare plasmids containing the nonsynonymous mutations of each protein gene, JFH1-Core-AM, JFH1-E2-AM, JFH1-NS3-AM, JFH1-NS5A-AM and JFH1-NS5B-AM, using the unique AgeI/BsiwI, Not I/Nsi I, Nsi I/BsrG I, and BsrG I/EcoR V restriction enzyme sites (Figure 2A). The sequence of JFH1-AM120 has been deposited in GenBank (Submission #1545284).

### HCV RNA Transfection

To generate the full-length genomic RNA, pJFH-1, pJFH1-AM120 was linearized at the 3′ end of the HCV cDNA with XbaI. The linearized plasmid DNA was purified and used as a template for T7 *in vitro* transcription (MEGAscript; Ambion, Austin, TX). *In vitro* transcribed RNAs above were transfected into cells by electroporation as described by Krieger et al [41], [42]. Briefly, trypsinized cells were washed twice and resuspended with serum-free Opti-MEM (Invitrogen) at the concentration of 1x10^7^ cells per ml. Ten micrograms of RNA were mixed with 0.4 ml of the cells in a 4-mm cuvette. A Bio-Rad Gene Pulser system was used to deliver a single pulse at 0.27 kV and 960 µF and the cells were plated in T75 Costar flasks (Corning). Transfected cells were cultured in complete DMEM for the times indicated in figure legends. Cells were passaged every 3 days; the presence of HCV in the corresponding supernatants was determined by immunofluorescence assays (IFA) for the NS5A proteins.

**Figure 3:**
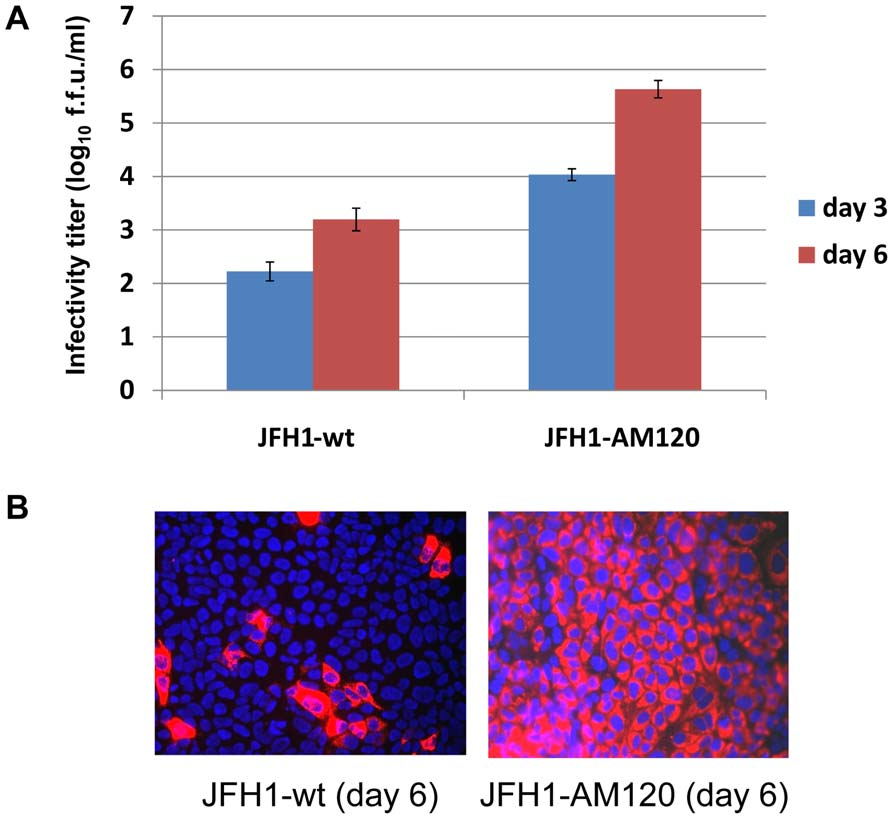
Effect of adaptation of HCV JFH1-AM120 on the production of infectious HCV particles. (Panel A) JFH1-AM120 RNA was electroporated into Huh-7.5 cells to produce the recombinant adapted virus in cell culture. The transfected cells were passaged every three days. The infectivity titers of the culture supernatants were measured (Methods). Viral titers are expressed as focus-forming units per milliliter (f.f.u./ml) assayed in triplicate and performed twice. The data are presented as mean ± standard deviation (n = 6). (Panel B) Titer of JFH1-AM120 virus compared to JFH1-wt. Huh-7.5 cells were inoculated with supernatants collected at six days post-transfection. Cells were fixed at 48h post-infection and infected cells were identified by NS5A-specific immunofluorescence (red) (Methods). Nuclear DNA was stained with DAPI (blue).

### Titration of Infectious HCV

The titer of infectious HCV was determined by immunofluorescence where the number of cell foci stained for the NS5A protein was directly visualized microscopically as described previously [42]. Briefly, cell supernatants were serially diluted 10-fold in complete DMEM. The supernatant was used to infect 1×10^4^/well naïve Huh-7.5 cells in 96-well plates. Inocula were incubated with cells for 2 hours at 37°C and then supplemented with fresh complete DMEM. The level of HCV infection was determined three days post-infection by immunofluorescence staining for HCV NS5A. The viral titer is expressed as focus-forming units per milliliter of supernatant (f.f.u./ml).

**Figure 4:**
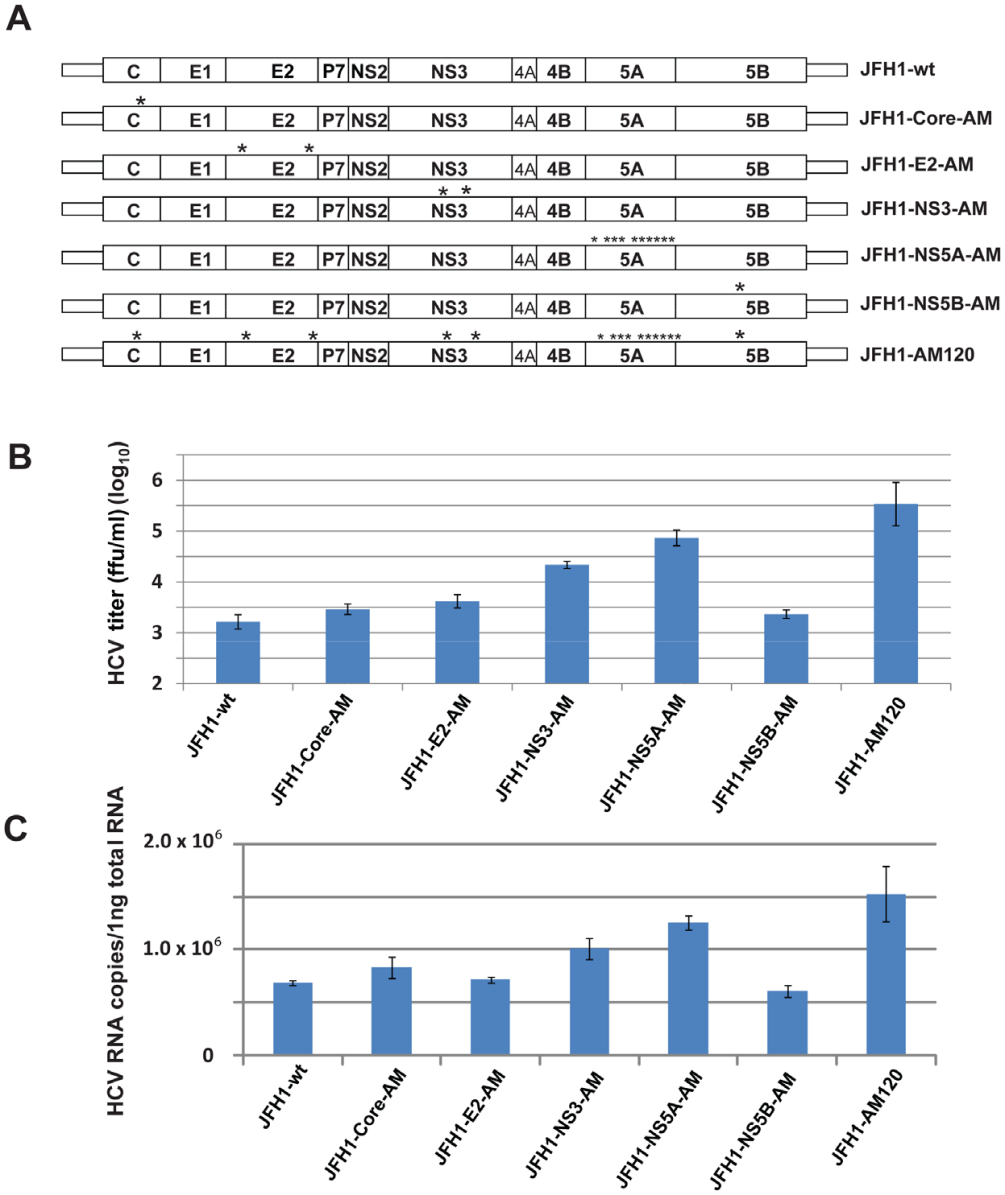
Effect of individual adaptive mutations on the production of infectious JFH1 HCV. (Panel A) Schematic of the wild-type (wt) and mutant JFH1 genomes. The location of HCV JFH1 adaptive mutations in the core, E2, NS3, NS5A and NS5B genes are indicated by * and are specified in Figure 2. (Panel B) Cell culture production of recombinant viruses with specific mutations. *In vitro* transcribed RNAs with specific mutations were electroporated into Huh-7.5 cells to generate recombinant mutant viruses. The transfected cells were passaged for six days. Infectivity titers of the culture supernatants were measured by titration assays. Viral titers are expressed as focus-forming units per milliliter (f.f.u./ml) (done in triplicate and performed twice). The data are presented as mean ± standard deviation (n = 6). (Panel C) HCV-RNA levels in cells six days after transfection. Intracellular-HCV-RNA levels were analyzed by quantitative RT-PCR. The mean ± standard deviations for two independent experiments are presented (qPCR assays, n = 6) (Methods).

### Immunofluorescence Assay (IFA)

Cells infected by HCV were washed with PBS, fixed with 4% paraformaldehyde, and permeabilized with 0.2% Triton X-100. Fixed cells were blocked with 1% bovine serum albumin and 1% normal goat serum in PBS. HCV NS5A protein was detected in cells by incubation with an NS5A-specific monoclonal antibody and visualized with the secondary goat anti-mouse IgG conjugated with Alexa Fluor 594 fluorescein (Invitrogen, 1∶1,000 dilutions). Cover slips were mounted onto slides with DAPI (Vector Labs) and the HCV proteins were visualized by fluorescence microscopy (Nikon E400).

**Figure 5:**
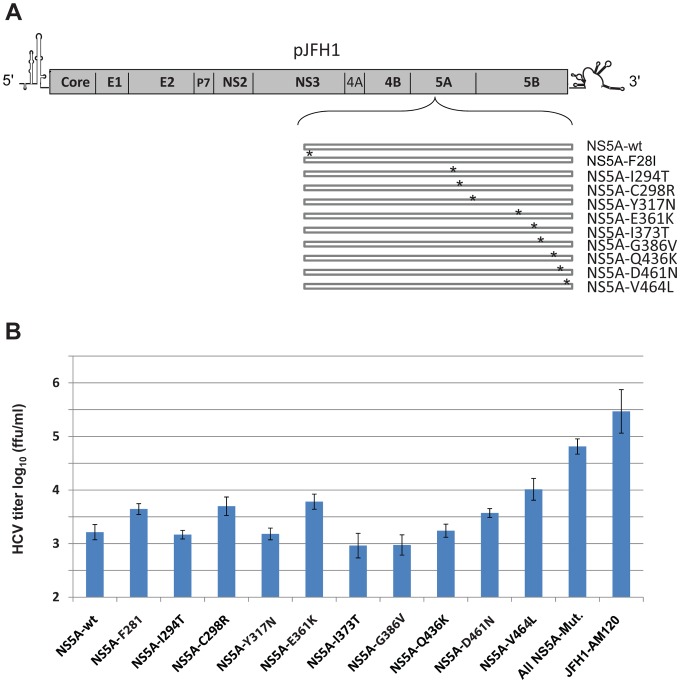
Effects of individual mutations in NS5A on the production of infectious cell culture JFH1 HCV. (Panel A) Schematic of the wild-type (wt) and individual mutant NS5A genomes. Adaptive mutations in NS5A are indicated by * and defined in Figure 2. (Panel B) Recombinant viruses with specific NS5A mutations. The *in vitro* transcribed RNAs were electroporated into Huh-7.5 cells. The transfected cells were passaged for six days, infectivity titers of cell culture supernatants were measured and expressed as focus-forming units per milliliter (f.f.u./ml) (done in triplicate and performed twice). The data are presented as mean ± standard deviation (n = 6).

### Quantification of HCV RNA

Total RNA was extracted by using Trizol (Invitrogen) according to the manufacturer’s instructions. One microgram of isolated RNA was reverse-transcribed by using a QuantiTect reverse transcription kit (Qiagen) with random primers. RT-qPCR analysis was performed as described previously [40]. HCV RNA was monitored by using the PCR primers 59-TCTGCGGAACCGGTGAGTA-39 (sense) and 59-TCAGGCAGTACCACAAGGC-39 (antisense). HCV transcript levels were determined relative to a standard curve comprising serial dilutions of plasmid containing the HCV JFH1 cDNA.

### Reporter Assay for Viral Replication and Infectivity

To determine the infectivity of culture medium containing reporter virus JFH1-AM120-Rluc, cell-free supernatant (centrifuged at 1500 g for 10 minutes) obtained from HCV RNA transfected cells was inoculated onto naïve Huh-7.5 cells in 24-well plates (in triplicate). At 48 hours post-inoculation the cells were lysed with 100 µl lysis buffer at room temperature for 15 minutes. *Renilla* luciferase activity was measured in cell lysates (20 µl) using a *Renilla* luciferase Assay System kit (Promega).

### Laser-scanning Confocal Microscopy

Cells transfected with JFH1-WT and JFH1-AM-120 were seeded onto 24 well plates with cover slips and cultured as described above. The cells were washed two times with PBS after 48 hours of culture, fixed with 4% paraformaldehyde, and permeabilized with 0.2% Triton X-100. Fixed cells were blocked with 1% bovine serum albumin and 1% normal goat serum in PBS. HCV NS5A protein was detected in cells by incubation with a NS5A-specific monoclonal antibody and visualized with a secondary goat anti-mouse IgG conjugated with Alexa Fluor 488 fluorescein (Invitrogen, 1∶1,000 dilutions). Neutral lipids present in lipid droplets were visualized by staining with LipidTOX Deep Red (Invitrogen). Cover slips were mounted onto slides with DAPI (Vector Labs) and slides were examined with a Zeiss LSM 510 Meta laser-scanning confocal microscope.

**Figure 6:**
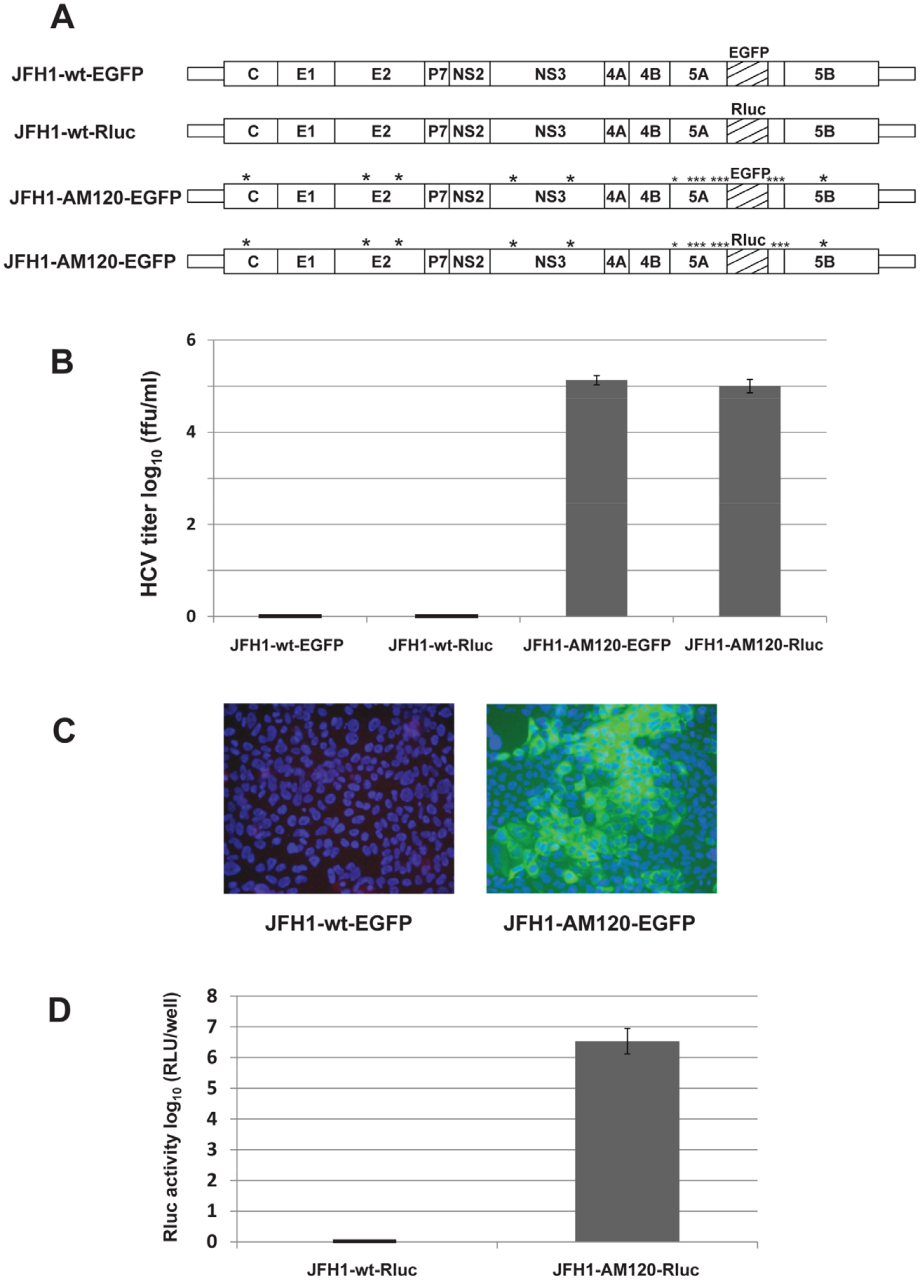
JFH1-AM120 HCV reporter viruses with gene inserts into NS5A; JFH1-AM120-Rluc and JFH1-AM120-EGFP. (Panel A) Schematics of the HCV JFH1-wt and JFH1-AM120 based reporter constructs. EGFP and *Renilla* luciferace (Rluc) genes were inserted in frame into a unique RsrII site located in the amino acid 397 codon of JFH1-wt and JFH1-AM120. (Panel B) *In vitro* transcribed RNAs were electroporated into Huh-7.5 cells. Transfected cells were passaged for six days, infectivity titers of culture supernatants were measured and viral titers are expressed as focus-forming units per milliliter (f.f.u./ml) (triplicates that were performed twice). The data are presented as mean ± standard deviation (n = 6). (Panel C) JFH1-AM120-EGFP virus compared to JFH1-wt-EGFP. Huh-7.5 cells were inoculated with supernatants collected at six days post-transfection. Cells were fixed at 48 h post-infection and infected cells were identified by fluorescence immunostaining and microscopy as in Figure 3. Nuclear DNA was stained with DAPI (blue). (Panel D) *Renilla* luciferase activity was measured in Huh-7.5 cells following infection with day six supernatants from cells transfected with JFH1-wt-Rluc and JFH1-AM120-Rluc RNA. Assays were done in triplicate and in two separate experiments. The data are shown as mean ± standard deviation (n = 6).

### Western Blot Analysis

The HCV-transfected Huh 7.5 cells were lysed in a radioimmunoprecipitation assay buffer (50mMTris–HCl, pH7.5, 150 mM NaCl, 1% Nonidet P40, 0.5% sodium deoxycholate) containing a cocktail of proteinase inhibitors (Roche). The total protein for each sample was measured with a standard protein assay (Bio-Rad). Twenty-five micrograms of total protein of each sample was analyzed by 8% SDS-PAGE and transferred to nitrocellulose membranes. The membranes were blocked with 5% skim milk. HCV proteins were detected with amonoclonal antibody specific for NS5A, horseradish peroxidase-conjugated goat anti-mouse immunoglobulin G (Bio-Rad) and a chemiluminescence substrate (Pierce). β- Actin was used as a control and was detected with an anti-β-actin monoclonal antibody (Sigma). The density of NS5A p56 and p58 bands were quantified using Image J software (NIH). P values were calculated using student’s t-test.

**Figure 7:**
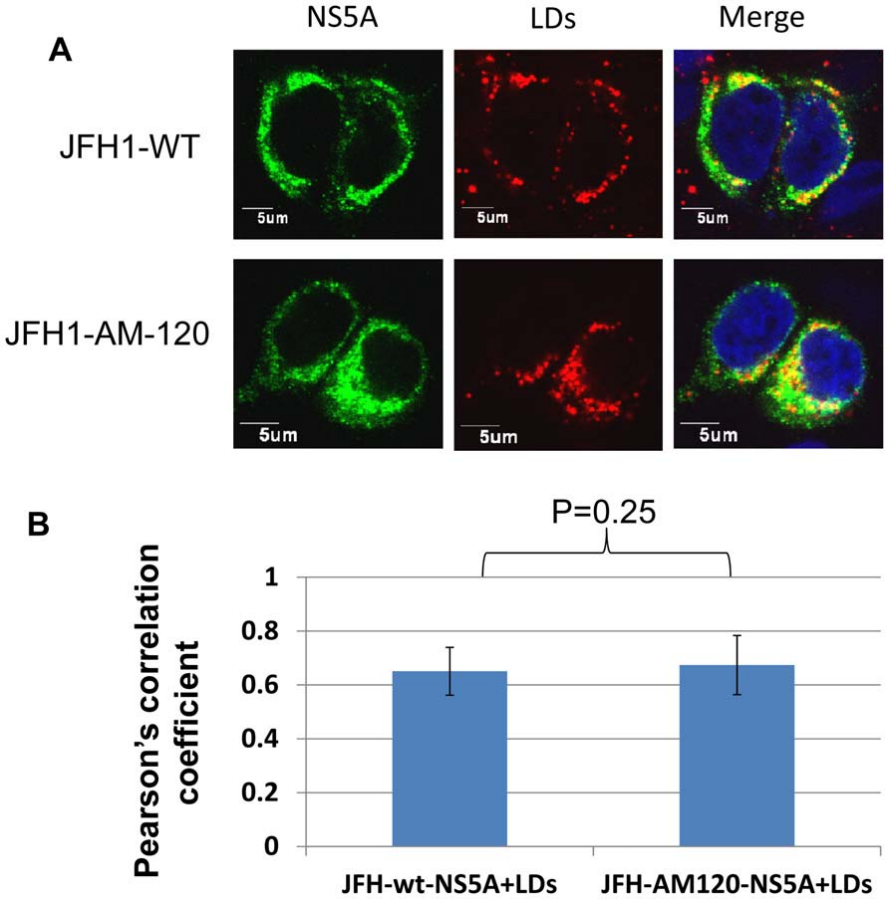
Co-localization of NS5A with lipid droplets (LDs). (Panel A) Cells transfected with JFH1-AM-120 and JFH-wt RNA were fixed after 48 hours of culture. LDs were stained with LipidTOX Red (red). The NS5A protein wasstained with an anti-NS5A antibody (green). (Panel B) Each triplicate sample had 50 cells analyzed using Image J software. The degree of co-localization was quantified and compared using Pearson’s correlation coefficients.

## Results

### Generation of Cell Culture-adapted JFH1-wt Virus

Infectious HCV particles were produced by electroporating *in vitro* transcribed genomic HCV JFH1 RNA into Huh-7.5 cells (Methods). Transfected Huh-7.5 cells were cultured and passaged as described in Methods. The titer of infectious virus particles released in the culture supernatants reached 5×10^4^ f.f.u./ml at 15 days. With the goal of generating higher titers of infectious viral particles, naïve Huh-7.5 cells (3×10^5^ cells per six-well plate) were infected at an m.o.i. of 0.2 and serially passaged every three days for a total of five passages (designated one cycle). At this time the cells had an atypical morphology suggestive of cell stress and culture supernatant was used to infect naïve Huh-7.5 cells. A total of eight passage cycles (120 days) were completed as described in Methods. The HCV titer was significantly increased by day 75 and reached 1×10^6^ f.f.u./ml indicating that JFH1-wt had acquired adaptive mutations that increased infectious virus production. Cells continued to be passaged and the virus titer was stable at approximately 1×10^6^ f.f.u./ml after another 45 days.At that time the passaging of cells was stopped and the adapted virus stock as named JFH1-AM120 (Figure 1).

**Figure 8:**
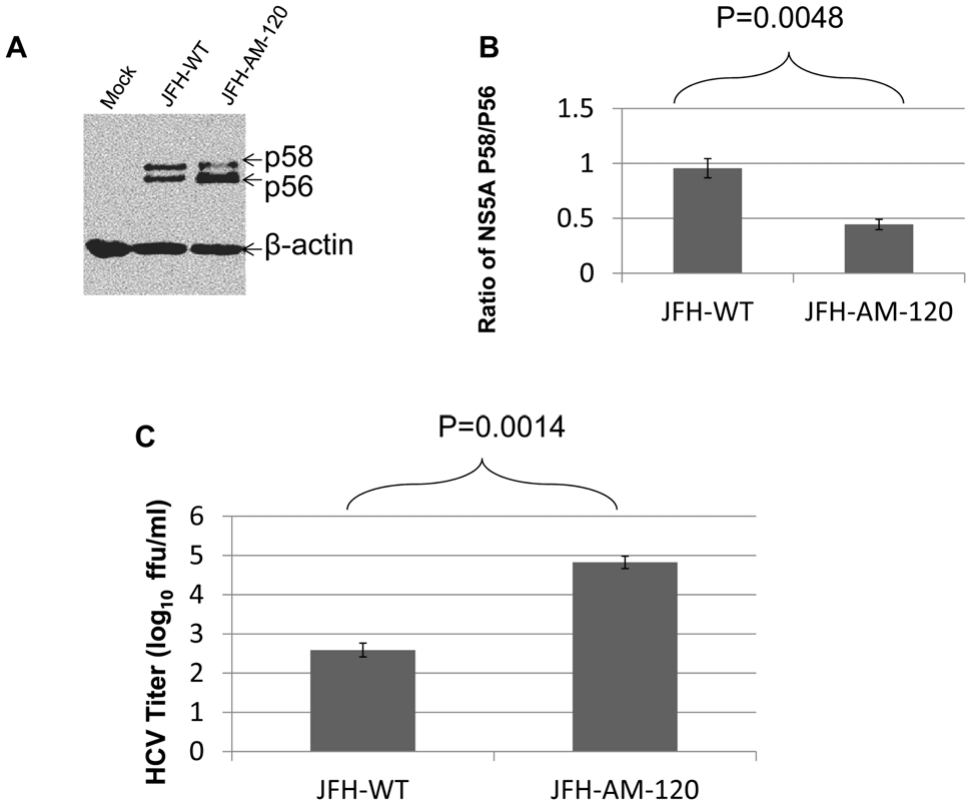
Phosphosphorylation of NS5A during HCV JFH1-WT and JFH1-AM-120 replication. Huh7.5 cells were transfected with JFH1-wt or JFH1-AM-120 RNA as described in Methods. (Panel A) After three days of culture cells were lysed for Western blotting using anti-NS5A and anti-β-actin bodies. Western blots of proteins separated by 8% SDS-PAGE gel were done as described in Methods and the p56 and p58 isoforms of NS5A were identified. (Panel B) The quantyof p56 and p58 were determined using Image J software and the ratios of p58/p56 are shown. (Panel C). Virus titers in cell culture supernatant collected at the same times post infection (48 hours) were measured by determining focus-forming units with NS5A Immunofluorescence assays (Methods). Assays were done in duplicate and performed four different times. The data arepresented as mean ± standard deviation (n = 8). P-values were calculated using the student’s t-test.

### Sequence Analysis of the Adapted JFH1-AM120

To identify the mutations responsible for enhanced production of infectious JFH1-AM120, HCV RNA isolated from infected cells was reverse transcribed and amplified by PCR in four fragments. The PCR fragments were subcloned to pJFH1 using unique restriction enzyme sites and the cDNA was sequenced as described in Methods (Figure 2A). A total of sixteen nonsynonymous mutations were found in JFH1-AM120. One mutation in the core and NS5B gene and two mutations in the E2 and NS3 genes were identified. The remaining ten mutations were found in the NS5A gene (Figure 2B).

### The Adapted Variant JFH1-AM120 Produces Higher Titers of Infectious HCV

To determine if these sixteen nonsynonymous mutations in JFH1-AM120 enhanced the production of infectious HCV, the RNA of JFH1-AM120 was transcribed *in vitro* and electroporated to Huh-7.5 cell. The cells were serial passaged and supernatant was collected after three and six days. JFH1-wt RNA was used as a control. The supernatants were assayed for titers of infectious HCV. The results provided evidence that JFH1-AM120 produced a higher titer of infectious virus in Huh-7.5 cells and rapidly reached 5.0×10^5^ f.f.u./ml by six days post-transfection. On the other hand, JFH1-wt RNA transfection only produced 1.5×10^3^ f.f.u./ml by the same time under the same conditions. The difference in titers between the two viruses was approximately 300 fold six days post-transfection (Figure 3A).

To further characterize the adapted virus produced from JFH1-AM120, Huh-7.5 cells were infected with supernatants of either JFH1-AM120 or JFH1-wt transfected cells and NS5A protein expression was measured by immunofluorescence. Interestingly, JFH1-AM120 spread much faster in cultured cells than JFH1-wt (Figure 3B). Nearly 100% of the JFH1-AM120 infected cells stained positive for NS5A after 48 hours, but only a few JFH1-wt infected cells stained positive. These results provide evidence that together the sixteen mutations in JFH1-AM120 enhanced the production of infectious HCV in cell culture.

### The Adaptive Mutations in NS5A of JFH1-AM120 Resulted in Higher Titers of Infectious HCV as Compared to Mutations in Other Proteins

Since mutations were found in Core, E2, NS3, NS5A and NS5B proteins (1, 2, 2, 10 and 1, respectively) we prepared plasmids containing the mutations of each protein to determine their individual effect on the production of infectious virus particles. PCR fragments with the mutations of individual proteins were subcloned into JFH1-wt to prepare constructs designated JFH1-Core-AM, JFH1-E2-AM, JFH1-NS3-AM, JFH1-NS5A-AM and JFH1-NS5B-AM as described in Methods (Figure 4A). RNA was transcribed *in vitro* from these plasmids and were electroporated in Huh-7.5 cells. At six days post-transfection the titer of infectious HCV and viral RNA were measured in supernatants. The results indicated the NS5A mutations had the greatest effect on the production of infectious HCV particles, with a titer of 4.7×10^4^ f.f.u./ml, and that the mutations in NS3 had the second greatest effect producing a titer of 4.0×10^4^ f.f.u./ml. Individually the other mutations in E2, NS3 and NS5B had little effect on HCV titers when compared to JFH1-wt. However, when all the mutations were present together, synergy was observed with JFH1-AM120 which produced a higher titer of infectious virus as compared with JFH1-NS5A-AM, 5×10^5^ f.f.u./ml and 4.7×10^4^ f.f.u./ml, respectively (Figure 4B). qPCR quantitation of copies of HCV RNA produced in the respective cell cultures showed similar results (Figure 4C). These results indicate that the changes in NS5A are largely responsible for the increase in infectious HCV produced in the adapted variant JFH1-AM120.

### Effects of Individual Mutations in NS5A on the Production of Infectious HCV

To determine which mutations in NS5A are responsible for the enhanced production of infectious HCV, recombinant genomes containing only one of the ten mutations were prepared (Figure 5A). The *in vitro* transcribed RNA synthesized from these and control plasmids were electroporated into Huh-7.5 cells to generate mutant and control viruses. Naïve Huh-7.5 cells were infected with cell supernatants obtained six days after transfection and the production of infectious HCV viral particles was measured by titration assays. Recombinant viruses with the single point mutations of F28I, C298R, E361K, D461N and V464L in NS5A significantly enhance the production of infectious HCV as compared to JFH1-wt (Figure 5B). However, the titers were lower than JFH1-5A-AM which had all ten mutations. This provides evidence that the mutations in NS5A have a synergistic or cooperative effect on the production of infectious HCV particles.

### Adaptive Mutations in NS5A Permit the Insertion of Reporter Genes and the Production of Infectious Chimeric Reporter JFH1 Viruses with Relatively High Titers

A unique restriction enzyme site in the NS5A C terminal region of JFH1-wt (RsrII, NS5Aaa 399) would be a convenient site to insert reporter genes. However, when the EGFP or *Renilla* luciferase (Rluc) gene was inserted into this position, no infectious chimeric reporter viruses were produced (Figure 6A, 6B). However, when EGFP or Rluc was inserted into the RsrII site of JFH1-AM120 and *in vitro* transcribed JFH1-AM120-EGFP or JFH1-AM120-Rluc RNA was electroporated into Huh-7.5 cells, infectious chimeric viruses were produced (Figure 6). The cells were serial passaged and supernatants were collected on the third and sixth day and assayed for infectious virus titers. JFH1-wt-EGFP and JFH1-wt-Rluc were used as controls. No infectious virus was detected from JFH1-wt-EGFP or JFH1-wt-Rluc. However, JFH1-AM120-EGFP and JFH1-AM120-Rluc produced relatively high titers of chimeric reporter viruses which reached approximately 1×10^5^ f.f.u./ml after six days post-transfection (Figure 6B). Cells were fixed at 48 hours post-infection and infected cells were visualized by fluorescence microscopy. EGFP fluorescence was only seen in JFH1-AM120-EGFP infected Huh 7.5 cells (Figure 6C). *Renilla* luciferase activity was only observed in JFH1-AM120-Rluc infected cells (Figure 6D).

#### Adaptive mutations were not necessary for the intracellular localization of NS5A on lipid droplets

Lipid droplets (LDs) have been reported to play a critical role in the process of intracellular assembly HCV (58). The recruitment of NS5A to LDs was shown to be a prerequisite for HCV assembly in host cells [58], [59]. To determine if the adaptive mutations in NS5A increased the assembly of HCV at this step, LDs and NS5A were stained in JFH1-wt and JFH1-AM-120 transfected cells and the co-localization of NS5A with LDs was analyzed. The data showed that the LDs were covered with NS5A in all infected cells (Figure 7A). The degree of co-localization of NS5A and LDs was quantified using Image J software and Pearson’s correlation coefficient analysis. No significant difference as observed between JFH1-wt and JFH1-AM-120 cells (Figure 7B) providing evidence that the adaptive mutations did not have an effect on the recruitment of NS5A to LDs and this step of virus assembly.

#### Decreased hyperphosphosphorylation of NS5A in the adapted JFH-AM-120

Previous studies showed that adaptive mutations which reduce NS5A hyperphosphorylation activate HCV replication. It was also proposed that a critical ratio between the p56 and p58 (hyperphosphorylated) phosphoforms of NS5A is required for optimal HCV RNA replication (62). We transfected Huh7.5 cells with HCV-wt and JFH-AM-120 RNA and after three days of culture, the cells were lysed for western blotting and cell culture supernatants harvested for HCV titer assays. The results showed a decrease in hyperphosphorylated NS5A (p58) and an increase in p56 in JFH-AM-120 as compared to JFH-wt (p<0.01) with a significant increase in titer of JFH-AM-120 as compared to JFH-wt (p<0.01) (Figure 8A.8B.8C). Previous studies have provided evidence that decreased hyperphosphorylation of NS5A (p58) increase HCV replication as discussed below.

## Discussion

Cell culture adaptation of viruses can enhance the production of infectious virus by a number of mechanisms, including shortening the replication cycle [17], [20]. For example, wild-type HAV replicates slowly and produces low yields of progeny virus, but continued passage in cell culture lead to progressive adaptation resulting in higher yields of infectious virus [43]–[45]. Such cell culture-adapted HAV variants were critical for the production of formalin-inactivated HAV vaccines which are now widely used [46], [47].

The establishment of an infectious HCV cell culture system with the JFH1 strain of genotype 2a HCV (or modifications of it) represented a major breakthrough in studying HCV [14]–[16]. Adaptive mutations that enhance the production of infectious virus particles from either wild-type JFH1 [17]–[20] or intergenotypic chimeras [15], [21]–[23], [48], [49] have represented further improvements. Nevertheless, the relatively low titers of infectious virus can be a limit in some applications.

In this report we identified sixteen new nonsynonymous mutations in the genome of a cell culture-adapted variant of JFH1, designated JFH1-AM120, that rapidly produces infectious titers of 5.0×10^5^ f.f.u./ml by six days RNA post-transfection as compared to JFH1-wt which produces only 1.5×10^3^ f.f.u./ml by the same time. JFH1-AM120 exhibited higher expression of the HCV proteins in cell culture than the wild-type virus. Unlike other adapted JFH1 viruses, JFH1-AM120 had ten of sixteen nonsynonymous mutations located in the NS5A gene. Analysis of mutations located in individual structural and nonstructural genes provided direct evidence that the mutations in NS5A had a major contribution to increasing the production of infectious virus by six days post-transfection with the titer reaching 4.7×10^4^ f.f.u./ml. However, mutations in the NS3 protein produced a titer of 4.0×10^4^ f.f.u./ml by the same time. JFH1-AM120, with all 16 mutations, produced the highest titers of infectious virus and reached 5×10^5^ f.f.u./ml at day six. These results provide further evidence that NS5A plays a major role in regulating the release of infectious virus particles in cell culture. One report has identified two different mutations in domain III of NS5A (E404D and T462I) that allowed reporter genes to be inserted into the NS5A region, albeit lower titers of infectious viruses were produced compared to our chimeric reporter viruses [37].

The involvement of domain I and domain II of NS5A in HCV RNA replication has been well documented [27], [29], [32], [33], [50]. Although the function of domain III is less understood, it was shown to be non-essential for competence of an HCV replicon [51]. More recently, domain III was reported to perform a critical role in the early phase of HCV assembly, as deletions or mutations severely reduced or abolished the production of infectious virus [30]–[32], [36], [52].

The NS5A mutations F28I, C298R, E361K, D461N and V464L that we report significantly enhanced the production of infectious HCV are in all three domains. E363K, D461N, and V464L are in domain III. This observation supports previous studies providing evidence that domain III has a critical role in HCV assembly. The F28I and C298R mutants we identified, located respectively in domain I and domain II, both enhance the production of infectious virus. These results provide evidence that domain I and domain II of NS5A plays a role in HCV assembly, which has not been previously reported.

Chimeric HCV reporter replicons and viruses are important tools for studying HCV biology and antiviral screening. Although HCV replicons have tolerated the insertion of reporter genes into the NS5A region, the insertion of reporter genes into this region of the cell culture infectious JFH1 genome generally ablates or reduces the production of infectious virions [53], [54]. Dicistronic JFH1 HCV-luciferase reporter viruses utilizing both the HCV and EMCV IRES have been an alternative approach to producing an infectious chimeric reporter virus [49], [55]. However, these systems have generally been limited for some applications because of the relatively low titers of infectious virus produced. The two chimeric reporter viruses we describe, JFH1-AM120-EGFP and JFH1-AM120-Rluc, rapidly produce relatively high titers of virus in Huh-7.5 cells and should expand the use of such reporter viruses. Moreover, the specific mutations we identified in JFH1-AM120 should aid the engineering of other infectious chimeric JFH1 viruses since the introduction of these mutations allowed HCV JFH1 to tolerate the insertion of relatively large foreign genes into the NS5A region without the need to delete the V3 or other genomic regions [42]. We have yet to determine why the mutations in JFH1-AM120 permit otherwise lethal gene insertions into the V3 region of NS5A to engineer infectious chimeric reporter viruses.

It has been proposed that HCV nonstructural proteins, including NS5A, and replication complexes are recruited to lipid droplet associated membranes by the core protein and that this is critical for the assembly of infectious virus [56]–[59]. Cholesterol and sphingolipid associated with HCV particles are also important for virion maturation and infectivity [57]. Our analysis of the co-localization of NS5A and lipid droplets showed no significant difference between JFH1-wt and JFH1-AM-120 transfected cells and provides evidence that the adaptive mutations in JFH-AM-120 did not alter this critical step of HCV replication.

NS5A exists as two phosphorylated forms, migrating as 56 and 58 kDa proteins in SDS-PAGE gels [60], [61]. The two major phosphoforms are also referred to as basally phosphorylated (p56) and hyperphosphorylated (p58) NS5A, respectively. Pulse-chase experiments have shown that the levels of p56 and p58 remain equal throughout the replication cycle of subgenomic replicon-containing cells [61]. A previous study showed that adaptive mutations which reduce NS5A hyperphosphorylation increase HCV replication. It was also proposed that a critical ratio between the two phosphoforms of NS5A is required for HCV RNA replication (62). Our analysis of the two phosphoforms NS5A showed a decrease in hyperphosphorylated NS5A in JFH-AM-120 compared to JFH-wt, indicating that that this change is involved in the mechanism of the increase in infectious HCV titer observed with the adapted JFH-AM-120.
