# Correction: A Cell Culture Adapted HCV JFH1 Variant That Increases Viral Titers and Permits the Production of High Titer Infectious Chimeric Reporter Viruses

**DOI:** 10.1371/annotation/2e81c9a3-4700-4869-aee9-f079092c77cd

**Published:** 2013-05-10

**Authors:** Shuanghu Liu, Li Xiao, Cassie Nelson, Curt H. Hagedorn

There was an error in the name of the fourth author.

The correct name is: Curt H. Hagedorn 

